# Could Self-Reported Body Sizes Be an Alternative Tool for Assessing Breast Cancer Risk in Postmenopausal Women?

**DOI:** 10.3390/ijerph19031809

**Published:** 2022-02-05

**Authors:** Beata Świątkowska, Marta Szkiela, Radosław Zajdel, Dorota Kaleta

**Affiliations:** 1 Department of Hygiene and Epidemiology, Medical University of Łódź, Żeligowskiego 7/9, 90-752 Łódź, Poland; marta.szkiela@umed.lodz.pl (M.S.); dorota.kaleta@umed.lodz.pl (D.K.); 2 Department of Computer Science in Economics, University of Łódź, POW 3/5, 90-255 Łódź, Poland; radoslaw.zajdel@uni.lodz.pl

**Keywords:** overweight, obesity, BMI, breast cancer, modifiable risk factor, pictogram

## Abstract

Background: The use of self-reported body size as an alternative tool to estimate body weight for health risk assessment is not widely reported, especially in relation to breast cancer. Therefore, we examined the association between breast cancer and body-mass index (BMI) and the usefulness of pictograms. Methods: The case–control study was conducted among postmenopausal women from 2015 to 2019. The study involved 151 women with breast cancer and 67 control subjects. Data were collected by a self-reported detailed questionnaire. Results: An increased, 4.13-fold risk of breast cancer (OR = 4.13; 95% CI [1.69, 10.28]) was observed for women with BMI 25.0–29.9 kg/m^2^ compared to women with normal BMI (18.5–24.9 kg/m^2^), whereas the association in the case of obese women was not statistically significant. An increased risk of breast cancer was observed for pictogram scores 3–4 (OR = 8.95; 95% CI [3.22, 24.88]) and for the highest level of self-reported body size, pictograms ≥ 5 (OR = 3.20; 95% CI [1.13, 9.09]). Conclusions: The risk of breast cancer is associated with an increased BMI and visual overweigh and obesity. The results suggest that a self-reporting alternative tool can be used to assess the prevalence of overweight/obesity, particularly in situations where no other tools are available.

## 1. Introduction

Overweight and obesity besides factors such as social history, childhood experiences, mental health, socioeconomic status are mainly caused by modifiable risk factors that can be reduced or controlled. Despite this, the number of overweight and obese people is growing all the time. Obesity is considered a top public health concern and the number of people with obese has almost tripled since 1975, worldwide [1]. According to WHO more than 1.9 billion adults (39%) were overweight and over 650 million (13%) were obese in 2016. Epidemiological studies have found that the prevalence of obesity increased from 8.9% to 14.8% in 2015 in women and from 5% to 10.1% in men. It was observed that the rise in the prevalence of overweight and obesity was always greater in women than in men, and was greatest between 1992 and 2002. The Non-Communicable Diseases (NCD) Risk Factor Collaboration (CD-RisC) estimated that by 2025, the prevalence of obesity will reach 21% for women and 18% for men [2]. 

According to a recently published report by the International Agency for Research on Cancer (IARC) Working Group, there are significant associations between BMI and risk of colon, rectum, gastric cardia, liver, gallbladder, pancreas, and kidney cancer and for esophageal adenocarcinoma. The IARC report also found that absence of body fatness lowers breast cancer risk in postmenopausal women, particularly for estrogen-receptor-positive tumors [3]. The World Cancer Research Fund (WCRF) confirmed the link between cancer, especially endometrium, esophagus, colon and rectum, liver, pancreas, postmenopausal breast and kidney cancer, and obesity [4]. A meta-analysis of 34 epidemiological studies involving over 2.5 million women has found that obese women have a higher risk of developing breast cancer after the menopause [5]. A few epidemiological studies have shown that obesity is a risk factor for breast cancer in postmenopausal women [6,7,8,9,10]. 

The biological mechanisms by which obesity may increase the risk of breast cancer are not fully understood. Hypothetical biological pathways include increased levels of endogenous hormones and inflammatory factors [11]. Obesity and a high content of adipose tissue result in an increased production of estrogens, the source of which in the period before the onset of menopause are the ovaries, and, in the postmenopausal period, mainly adipose tissue. 

In health-related research, self-reported body size and pictorial representations ranging from very lean to overweight is sometimes used to describe the characteristics of the human body and provides an alternative approach to estimate body weight and height. It is especially useful when studies do not have precise measuring instruments or do not have data on anthropometric measures in the past. Results from the analysis of pictogram body-size score, as an alternative measure of excess body weight, showed that the body image pictogram is a good semi-quantitative tool for BMI estimation [12]. The use of this tool for health risk assessment is not widely reported, especially in relation to breast cancer. 

The aim of this study was to examine the relationship between breast cancer and BMI and the usefulness of pictograms in the risk assessment of breast cancer in postmenopausal women. 

## 2. Materials and Methods

### 2.1. Study Design and Patients Selection

The case–control study was conducted among postmenopausal women in the Lodz Province during the period from 2015 to 2019. The case group included 151 women diagnosed with breast cancer. The women were patients of the oncological surgery department and clinic. 

The criterion for including women in the case group were histologically confirmed breast cancer and no history of other cancers.

The control group included 67 women without a diagnosis of breast cancer. Controls were community-based. The criterion for including women in the control group was no history of any cancers.

### 2.2. Methods

A detailed questionnaire was used to determine demographic variables, anthropometric data, lifestyle and reproductive history characteristics (smoking status, alcohol consumption, age of menarche, number of pregnancies, age of first birth, duration of breastfeeding), family history of cancer, and employment history.

#### Anthropometric Data

Detailed information was collected on body size. Subjects were asked to report their weight (kg) and height (m). Then, based on these data, we calculated body-mass index (BMI) with the following formula: weight (kg) divided by height squared (m^2^). BMI was analysed in three variables: 18.5–24.9 kg/m^2^ (normal), 25.0–29.9 kg/m^2^ overweight) and ≥30 kg/m^2^ (obese). 

**Figure 1:**
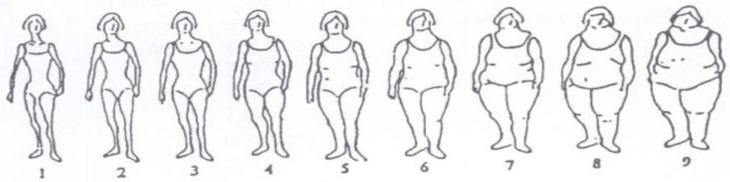
Pictogram for body size.

We also asked the respondents to assess the individual’s perception of their body size on the pictogram. A total of nine different body sizes (pictogram) were used to indicate body sizes (from very lean to obese). The analysis covered two time points (assessed by women 15 years before the interview and the period when the data were collected) (Figure 1). We used pictograms resembling those published earlier and used in various epidemiological studies. Pictogram scores 1 and 2 were assigned to participants with normal weight, pictogram scores 3–4 were selected by subjects with overweight, and finally, pictograms equal or higher than 5 were selected by people with obesity.

### 2.3. Compliance with Ethics Guidelines

The study was approved by the ethics boards of all participating institutions and participants provided written informed consent. The study design received a positive opinion from the Bioethics Committee at the Medical University of Lodz (RNN/236/15/EC on 22 September 2015).

### 2.4. Statistical Methods

The results were analysed with the use of statistical methods, including some multidimensional tests. The Shapiro–Wilk test was used to assess normal distribution. The studied characteristics of non-normal distribution as well as qualitative and quantitative data were analysed with the use of non-parametric tests, including, Pearson’s chi-squared and Mann–Whitney U tests. General descriptive statistics methods were also used. A logistic regression model was used for multivariate analysis, where applicable. Analysis of covariance (ANCOVA) was used to control for the effect of some continuous variables that were not of primary interest when evaluating the effect of categorical independent variables on the dependent one. Goodness of statistical fit of the models was assessed with Hoshmer–Lemeshow test and ROC curves. The statistically significant *p* level was at <0.05. The program used was Statistica 13.3, licenced by the Medical University of Lodz, Poland.

## 3. Results

A total of 151 breast cancer cases and 67 controls were included in the analysis. The characteristics of the examined women are presented in Table 1. The mean age in the case group was 62 years while in the control group it was 59. Control tended to be more highly educated than case; percentage of controls with higher education was 16% (9% among cases) and with secondary education was 46% (38% among cases). More controls than cases lived in rural and small cities areas. About 64% of controls never smoked cigarettes as compared with 33% of cases. In the case group, a higher percentage of women were current (25%) or former smokers (42%) than in controls (16% and 19% respectively). Among cases, the mean age of first menarche and age of first birth were lower than in the control group. The mean duration of breastfeeding, both in the case group and in the control group, was 5 months. A total of 29% of cases have a family history of cancer compared with 3% of controls.

Cases generally had a higher body-mass index than non-cases and the difference was statistically significant. The average BMI value in the control group was 24 kg/m^2^, while in the case group it was 26 kg/m^2^. The proportion of controls who had normal weight was 70% compared to 40% of controls. In the case group there was a significantly higher percentage of people with overweight (35% vs. 16% among controls) and obesity (25% vs. 13% among controls). Among cases, 36% showed pictograms score < 3, 41% pictograms 3–4 and 23% pictograms ≥5. In the control group, most women (81%) had a score of 3 or less. The relative frequency of pictogram score 5 or more was substantially higher in cases (23%) than in controls (10%), while scores 1–2 were more common among controls (81%) than among cases (36%).

Unadjusted and adjusted analyses of the associations between BMI and breast cancer risk are presented in Table 2. Our results for BMI suggest an increased breast cancer risk in postmenopausal women. In unadjusted analyses, BMI was found to have 1.13 (95% CI [1.05, 1.21]) risk of breast cancer. An increased, 3.71-fold risk of breast cancer (OR = 3.71; 95% CI: [1.75, 7.88]) was observed for women with BMI 25.0–29.9 kg/m² compared to women with normal BMI (18.5–24.9 kg/m^2^). Among obese patients there was a significant positive association with an OR of 3.17 (OR = 3.17; 95% CI: [1.130, 7.21]) (Table 2). 

Three variables were found to be significant on the univariate analysis, which included smoking, family history of breast cancer and earlier age of first childbirth. Age of patients, place of residence, education level, number of children, time of breast feeding, date of menarche as well as age of menopause were not found to be statistically significant confounders (*p* > 0.05). Multivariate analysis using all variables which was found to have a significant risk on univariate analysis and showed that the probability of breast cancer increased from 313% (OR = 4.13; 95% CI [1.69, 10.28]) in overweight women to 75% (OR = 1.75; 95% CI [0.68, 4.50]) in obese women, whereas the association in the case of obese women was not statistically significant. The association between higher BMI and the risk of breast cancer was stronger among overweight women, than the number of patients categorized as obese decreased, and the association became not significant.

Furthermore, we examined associations between self-reported body sizes (pictogram) and risk of breast cancer in postmenopausal women. The analysis covered two time points (assessed by women 15 years before the interview and the period when the data were collected). No positive association was observed for body size assessed by women, therefore the analysis was presented for the self-reported body sizes 15 years ago. The results obtained from multiple logistic regression analysis are shown in Table 3. In general, the data suggest that self-reported body-size pictograms were associated with increased risk of breast cancer in premenopausal women. More specifically, compared to the lowest category (scores < 3), an increased risk of breast cancer was observed for pictogram scores of 3–4 (OR = 8.95; 95% CI [3.22, 24.88]) and for the highest level of body size, pictograms ≥ 5 (OR = 3.20; 95% CI [1.13, 9.09]). The data indicated that women with early first childbirth were found to have a 10% lower chance of developing breast cancer (OR = 0.90; 95% CI [0.85, 0.98]). Women with a family history of breast cancer were found to be 7.87 times (95% CI [1.43, 43.22]) more at risk of breast cancer compared to those no with family history of breast cancer. Tobacco smoking was also found to have a significant positive association with an OR of 5.05 (95% CI [2.08, 12.30]) for smokers at any time and an OR of 2.89 (95% CI [1.10, 7.58]) for current female smokers (Model 1).

When we restricted the analysis to self-reported body size as a continuous variable (Model 2) we observed a 38% increased risk (OR = 1.38; 95% CI [1.10, 1.72]) of breast cancer per unit increase in body-size score assessed 15 years before the interview. The other parameters taking into account variables that were found to have a significant risk were smoking, family history of breast cancer and earlier age of first childbirth, which showed a similar level of risk in the analysis using pictograms as categorical variable (Model 1) (Table 3).

## 4. Discussion

Overweight and obesity are commonly known modifiable risk factors of non-communicable diseases, including cancers. In our study, we observed a 3.71-fold higher risk of breast cancer in overweight women and a 3.17-fold higher risk of breast cancer in obese women compared to women who were of normal weight. Current epidemiological studies confirm an association between obesity and an increased risk of postmenopausal breast cancer. In epidemiological studies, it has been also observed that obesity is associated with larger tumors, positive lymph nodes and shorter overall survival [13,14]. The American Cancer Prevention Study II, conducted from 1982–1998 among 495,477 women, found that women who had a BMI > 40 kg/m^2^ had more than a 2-fold higher risk of death compared to women of normal weight (RR = 2.12; 95% CI [1.41, 3.19]) [15]. In the Women’s Health Initiative study conducted from 1993–1998 with a follow-up in 2010, among 67,142 postmenopausal women, an increase of breast cancer risk was found in women with a higher BMI (>35) compared to women of normal weight [16]. In a Korean case–control study conducted from 2003–2010 among 16,190 women with breast cancer and twice as large a control group, it was found that the breast cancer risk among obese women is higher than in women of normal weight. The increased risk of breast cancer depends on its type, in postmenopausal women: Luminal A (OR = 2.35, 95% CI [2.01, 2.75]), Luminal B HER2 negative (OR = 1.81, 95% CI [1.46, 2.25]) and triple-negative subtype (OR = 2.25, 95% CI [1.85, 2.72]), in premenopausal women: triple-negative subtype (OR = 1.60, 95% CI [1.27, 2.02]), Luminal A (OR = 1.24, 95% CI [1.06, 1.45]) and HER2 express subtype (OR = 1.43, 95% CI [1.26, 1.62]) [17]. Cabat et al., based on a prospective cohort study, concluded that obesity, regardless of metabolic health, was associated with increased breast cancer risk. Obesity and metabolic disorders were associated with the highest risk (HR = 1.62; 95% CI [1.33, 1.96]) [18].

Chlebowski et al. presented a different approach to the problem of the relationship between BMI and breast cancer risk. A study among postmenopausal women found that women who lost weight had a significantly lower breast cancer risk compared with women whose weight remained stable (HR, 0.88; 95% CI [0.78, 0.98]) [19]. In an analysis of the study conducted in 2005–2014, among 30,109 Asian women, it was found that postmenopausal women with increased BMI ≥ +5.0 had a significantly higher risk of breast cancer (HR = 1.902; 95% CI [1.202, 3.09]) than women whose BMI did not change significantly (−/+ 2.5) [20].

Previous studies have shown that pictograms are a good semi-quantitative tool for BMI estimation. Peterson et al. examined body perception among 215 students. The results provided data that using pictograms may help health programs track and measure body image perception data among the population [21]. In a study by Williamson, the credibility and validity of the research tool called Body Image Assessment for Obesity (BIA-O) was confirmed [22]. In current epidemiological studies, pictograms are mainly used to assess childhood body size. The strong point of our extensive approach was the use of a combination of traditional body-mass index assessment and an alternative tool and a relatively large postmenopausal women group. The limitations of our study were the self-assessment questionnaire and anthropometric self-assessment. The results of our study showed that self-reported body-size pictograms were associated with increased breast cancer risk in postmenopausal women. Compared to the lowest category (scores < 3), an increased risk of breast cancer was observed for pictogram scores of 3–4 (OR = 8.95; 95% CI [3.2, 24.88]) and for the highest level of body size, pictograms ≥ 5 (OR=3.20; 95% CI [1.13, 9.09]). In addition, several factors, such as smoking, age of first childbirth, and family history of breast cancer were studied as potential confounding factors. We also found an association between self-reported body size as a numerical variable with 38% increased risk (OR = 1.38; 95%CI [1.10, 1.72]) of breast cancer per unit increase in body-size score.

## 5. Conclusions

Postmenopausal women with overweigh and obesity are at increased risk of breast cancer. Our findings showed that self-reported body size may be used as a quick and effective alternative to measured BMI, which is essential for the detection of this modifiable risk factor for breast cancer. The results suggest that a self-reporting alternative tool can be used to assess the prevalence of overweight/obesity, particularly in situations where no other tools are available.

Healthcare professionals have an easy tool to assess the risk of health problems that can be linked to the risk of breast cancer, which has important implications in preventive actions. As our results may not be generalized to other populations, there is a need for more studies comparing BMI measurements and self-reported body size.

## Figures and Tables

**Table 1:** Characteristics of study population.

Variable	Controls (*N*, %)	Cases (*N*, %)	*p*-Value
Population	67 (30.7)	151 (69.3)	
Age (median; IQR)	58.6 (10.4)	61.6 (1.8)	0.052
Education			
primary	25 (37.3)	80 (52.9)	0.033
secondary	31 (46.3)	57 (37.7)	0.237
higher	11 (16.4)	14 (9.3)	0.126
Place of residence (number of inhabitants)			
rural	27 (40.3)	50 (33.1)	0.306
cities < 50th	19 (28.4)	30 (19.9)	0.166
cities 50–100th.	16 (23.8)	37 (24.5)	0.922
cities > 100th.	5 (7.5)	34 (22.5)	0.007
Smoking			
never	43 (64.2)	50 (33.1)	<0.0001
past	13 (19.4)	64 (42.4)	0.001
current	11 (16.4)	37 (24.5)	0.184
Age at menarche (median; IQR)	14.0 (1.0)	13.0 (2.0)	0.015
Age of first birth (median; IQR)	22.4 (5.7)	21.6 (4.0)	0.193
Duration of breastfeeding (months, median; IQR)	5.0 (4.0)	5.0 (2.0)	0.785
Family history of cancer			
no	2 (3.0)	44 (29.1)	<0.0001
yes	65 (97.0)	107 (70.9)	<0.0001
BMI (median; IQR)	23.7 (4.4)	26.3 (6.0)	<0.0001
BMI categorical (kg/m^2^)			
18.5–24.9	47 (70.2)	61 (40.4)	<0.0001
25.0–29.9	11 (16.4)	53 (35.1)	0.005
≥30	9 (13.4)	37 (24.5)	0.065
Pictogram for body size (continuous)	2.0 (1.7)	3.2 (1.8)	<0.0001
Pictogram for body size (categories)			
<3	54 (80.6)	54 (35.7)	<0.0001
3–4	6 (8.9)	63 (41.7)	<0.0001
≥5	7 (10.4)	34 (22.5)	0.035

**Table 2:** Associations of BMI and breast cancer risk among postmenopausal women.

Parameters	Model 1 ^a^		Model 2 ^b^	
	OR	95% CI	OR	95% CI
BMI categorical (kg/m^2^)				
18.5–24.9	1.00	-	1.00	-
25.0–29.9	3.71	1.75–7.88	4.13	1.69–10.28
≥30	3.17	1.39–7.21	1.75	0.68–4.50

Abbreviations: BMI—body-mass index; OR—odds ratio; CI—confidence interval; ^a^ Unadjusted; ^b^ Adjusted for age, smoking, age of first childbirth, family history of breast cancer.

**Table 3:** Associations of self-reported body size and breast cancer risk among postmenopausal women.

Parameters	Model 1 ^a^		Model 2 ^b^	
	OR	95% CI	OR	95% CI
Pictogram for body size (categories)				
<3	1.00	-	-	-
3–4	8.95	3.22–24.88		
≥5	3.20	1.13–9.09		
Age of first childbirth (continuous)	0.90	0.85–0.98	0.92	0.86–0.98
Family history of breast cancer				
no	1.00	-	1.00	-
yes	7.87	1.43–43.22	8.27	1.71–40.13
Smoking				
never	1.00	-	1.00	-
ever	5.05	2.08–12.30	3.98	1.69–9.36
current	2.89	1.10–7.58	2.56	1.03–6.35
Pictogram for body size (continuous)	-	-	1.38	1.10–1.72

Abbreviations: OR—odds ratio; CI—confidence interval; ^a^ All variables except self-reported body size as a continuous variable were included in the multivariate model; ^b^ All variables except self-reported body size aggregated into categories were included in the multivariate model.

## Data Availability

The data presented in this study are available on request from the corresponding author. The data are not publicly available due to privacy restrictions.
